# Numerical Investigation on the Transverse Vibration of Prestressed Large-Span Beams with Unbonded Internal Straight Tendon

**DOI:** 10.3390/ma14092273

**Published:** 2021-04-28

**Authors:** Mohammad Reza Ghaemdoust, Feiliang Wang, Siping Li, Jian Yang

**Affiliations:** 1 State Key Laboratory of Ocean Engineering, Shanghai Jiao Tong University, Shanghai 200240, China; mohammad.ghaemdoust@sjtu.edu.cn (M.R.G.); j.yang.1@sjtu.edu.cn (J.Y.); 2 Shanghai Key Laboratory for Digital Maintenance of Buildings and Infrastructure, School of Naval Architecture, Ocean and Civil Engineering, Shanghai Jiao Tong University, Shanghai 200240, China; 3 School of Civil Engineering, University of Birmingham, Birmingham B15 2TT, UK

**Keywords:** prestressed large-span beam, fundamental natural frequency, dynamic behavior, vibration, eccentricity, moving load

## Abstract

This paper deals with the effect of the prestress load on the free and forced dynamic behavior and vertical vibration of the prestressed beams. The analysis applies both the analytical frequency equation and the finite element method (FEM) using ABAQUS software to predict the fundamental natural frequency (FNF) of the simply supported unbonded prestressed beams. The energy method has been employed to derive the effective prestressing load to determine the eccentricity effect. In regard to the forced response of the prestressed beam, a moving point load with a constant value and various velocities and excitation frequencies is applied. Extensive parametric studies are carried out taking into account different factors including prestress load, eccentricity, concrete ratio, span-to-depth ratio, velocity, and frequency of the moving load. The comparison of the FNFs obtained by the formula with those obtained from FEM models indicates that the results are in a good agreement. This convergence demonstrates that the proposed formulation can predict the FNF of the eccentrically prestressed beams with high reliability. The time-histories curves for midspan displacement of the unbonded prestressed beams and the dynamic magnification factors are also evaluated. The results illustrate that the aforementioned factors have an indispensable contribution to the beam dynamic behavior.

## 1. Introduction

Prestressed beams have been extensively used in large-span bridges and different types of structures over the course of recent years. An increasing span of a girder makes a bridge heavier, which results in less sustainable conditions. In order to address this drawback, prestressing strands are employed in the structure to bear a proportion of the loads and structure’s weight for the sake of a reduction in the material consumption and weight of structure as a result. The beams prestressed with high-strength cables offer major advantages including elastic behavior under heavier loads, a higher ultimate capacity, reduced weight, and enhanced fatigue behavior. Prestressing can be used for strengthening an existing structure and in a new construction as well [1]. There are two conventional pre-tensioning schemes: draped and straight tendon; the former scheme improves ductility more than the latter. However, due to having lower construction expenses, straight tendons are more favorable [2].

Several studies have focused on the importance of the prestress load effect on the dynamic behaviors and more specifically on the fundamental natural frequency (FNF) of such beams. Kerr [3] carried out a numerical and experimental investigation on the dynamic response of a prestressed beam. It was concluded that concentric axial prestressing load does not change the bending deflection, and particularly the natural frequencies of the beam. Dall’Asta and Dezi [4], using three-dimensional theory, reported that the intensity of the force has a negligible impact on the natural frequencies of the prestressed beam. Deák [5] believes that for uncracked units, the natural frequencies are not seriously altered. Jain and Goel [6] inferred that the prestress force is an internal force. Therefore, they denied the alteration in the natural frequencies due to the “compression softening” effect. Based on mathematically derived governing equations, Hamed and Frostig [7] stated that the natural frequencies of the beam do not change due to prestress force for either bonded and unbonded tendons. Bonopera et al. [8,9,10] have done extensive experimental studies on the free transverse vibration of a prestressed concrete beams subjected to different levels of prestressing. They determined that the FNF of the beam remains unaffected and is not a suitable parameter for prestress loss estimation. Moreover, it was shown that the alteration of the concrete initial elastic modulus influences the fundamental frequency of the uncracked beams. Noble et al. [11,12] have done an extensive experimental program on the dynamic behavior of the rectangular hollow sections (RHS) and reinforced concrete beams to study the relationship between natural frequency and prestressing load. They expressed that the “compression softening” theory is invalid for the post-tensioning load, since it differs from an axial compressive load. Thus, they concluded that the FNF does not change after exerting prestressing load.

On the other hand, Raju and Rao [13] indicated that application of a prestress load as an external axial force reduces the lower modes of natural frequencies. Miyamoto et al. [14] conducted numerical and experimental studies on the dynamic characteristics of the externally prestressed bridges. Several parameters including prestress load level and eccentricity were considered. It was concluded that prestressing affects the natural frequencies depending on the tendon arrangement. In the presence of small amounts of eccentricity, the natural frequency tends to decrease because the axial force is predominant. However, this effect is reversed when the eccentricity is large enough. The research conducted by Law and Lu [15] illustrates that the natural frequencies of a simply supported beam decrease in the presence of prestress load. It was also found out that the low-order natural frequencies are more sensitive to the “compression softening” effect. In the investigation carried out by Jaiswal [16], the influence of prestressing force on the FNFs of beams with bonded and unbonded tendons was studied. It was indicated that the FNFs of the beams with unbonded tendons significantly vary with regard to the prestressing load and eccentricity level, but they do not change for the beams with bonded tendons.

Prestressed concrete structures may develop cracks resulting from the natural environment [17]. Monitoring the natural frequency is one of the approaches used to investigate the effect and severity of the cracks on a beam performance. A two-phase experimental and numerical investigation by Elshamy et al. [18] illustrated that the crack location and depth are the most influential factors, since they can reduce the stiffness and consequently the natural frequency. Hamed and Frostig [19] implemented nonlinear material properties for concrete and prestress strand to model the crack effect and geometric properties in their proposed incremental formulation to investigate the natural frequencies of tendon-bonded reinforced concrete beams. Different crack sizes were defined to calculate the natural frequencies. It was understood that the FNF reduced drastically as a result of cracks in the concrete and the corresponding mode shape was half-sine bending in all cases. Saiidi et al. [20] studied the possibility of the prestress loss detection of the prestressed members using vibration frequencies. They indicated that the existence of prestress force brings about the closure of microcracks of the concrete and consequently increases bending rigidity, which results in the rise in the fundamental frequency. Likewise, Gan et al. [21] concluded that the natural frequencies of a concrete beam increase as a result of closing the cracks inside the concrete beam. The results of an experiment on the dynamic behavior of a prestressed concrete beam showed that the natural frequency increases following eccentric prestressing of load [22].

Several investigations have tried to find a solution for calculating the dynamic response of the simply supported beam under a moving load [23,24,25,26]. Dynamic vibration of the axially loaded beams resting on the elastic foundation along with relevant factors including the variable elastic foundation, magnitude of vibration and axial load, and boundary conditions were studied by Mirzabeigy and Madoliat [27]. It was shown that the nonlinear frequency increases when the elastic foundation has a distribution close to the fundamental eigenmode. The influence of stiffness of Winkler elastic and number of layers on the dynamic response of the multi-layered simply supported beams subjected to a moving mass was investigated by Hashemi and Khaniki [28]. The experimental and numerical study on the nonlinear behavior of a slender beam subject to the various forcing amplitude showed the reliance of the resonance on the force amplitude [29]. Law et al. [30] have proposed an approach for identifying prestressing force based on the bridge-vehicle system. Application of a force identification technique for a prestressed bridge has shown that the static bending moments on a prestressed concrete beam are larger due to the softening effect [31]. Şimşek and Kocatürk [32] investigated the dynamic behavior of a simply supported prestressed beam under a moving harmonic load. Their numerical investigation included eccentricity and nonlinear geometry effects. It was explained that the response of the beam increases as a result of reduced bending stiffness, which is attributed to the “compression softening”. Kumar and Saleeb [33] have modeled a number of large-scale examples with infinite sliding between moving load and the structure to clarify contact interaction capabilities of finite element analysis (FEA) software. To the best of the authors’ knowledge, there are only a few papers that have dealt with the dynamic response problem associated with a moving load using FEA software.

A few studies have been conducted to examine the relationship between prestress force and dynamic behavior of the beams. As it evidently a controversial issue, scholars have presented different solutions. Therefore, there is no determined conclusion on the effect of prestress force on the natural frequency of the large-span beams. Furthermore, there is a scarcity of practical formulas that are able to calculate the natural frequency of eccentrically prestressed beams. For this paper, the vertical vibration behavior of unbonded prestressed large-span beams was investigated. To begin with, a formulation to estimate natural frequency which includes eccentricity impact was derived. Then, a thorough simulation with the aid of ABAQUS considering several factors was conducted to discuss the dynamic characteristics of the abovementioned beams. Concerning the span-to-depth ratio effect, different beam lengths were selected. A number of examples are presented here with the aim of detecting the effects of prestress reinforcement on the free and forced vibration response of prestressed beams.

## 2. Natural Frequency of the Eccentrically Prestressed Beam

Natural frequencies of axially loaded beams are available in many references, see for example [34]. However, there is a necessity for a practical solution that deals with the natural frequencies of eccentrically prestressed beams. To investigate the effect of prestress force on the beam’s natural frequencies, it was supposed that the behavior of the beam follows Euler–Bernoulli’s beam theory. According to this theory, the plane sections remain, are normal to the longitudinal axis, and the rotation of cross-sections and shear effect of the beam are neglected. The Euler–Bernoulli theory is valid for the beams with a high length-to-depth ratio [34]. The transverse vibration of a prestressed beam by external tendons in absence of transverse force using the extended Hamilton’s principle can be expressed as follows:

(1) $$EI_{eq}\frac{\partial^{4}y}{\partial x^{4}}+\rho A\frac{\partial^{2}y}{\partial t^{2}}+P\frac{\partial^{2}y}{\partial x^{2}}=0$$

where *EI_eq_* is the flexural stiffness of the beam with tendons, *ρ* is the density of the prestressed beam with a tendon, A is the cross-sectional area of the beam and the tendon, and *P* is the axial compressive force. Using the method of separation of variables, a solution of Equation (1) can be found for *x* and *t*:

(2) y(x,t)=Y(x)(Acosωt+Bsinωt)

By substituting Equation (2) into Equation (1), we found:

(3) $$EI_{eq}\frac{d^{4}Y}{dx^{4}}+P\frac{d^{2}Y}{dx^{2}}-\rho A\omega^{2}Y=0$$

### Proposed Effective Prestress Load Due to Eccentricity

The total external work of a set of point forces acting on a linear elastic structure causing displacements is stored as the strain energy. In a system with no energy dissipation, this external work is equivalent to the strain energy. The total strain energy over an entire volume of a solid elastic element, can be written as:

(4) $$U=\frac{1}{2}\int_{V}{\left\{\sigma \right\}}^{T}\left\{\varepsilon \right\}dV\;$$

**Figure 1:**
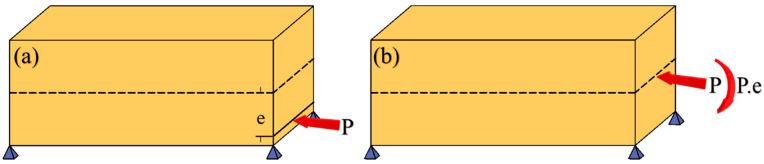
(**a**) Simply supported beam with eccentric axial load, (**b**) the equivalent transferred eccentric compressive load as a centric compressive axial load and a moment.

Assume a simply supported beam is subjected to an eccentric force P with length l, as shown in Figure 1.

Assuming constant axial force along the beam length, the total strain energy of the beam in Figure 1 subjected to a bending moment and a compressive force is computed as:

(5) $$U_{tot}=\frac{1}{2}\int_{l}\left(\frac{P^{2}}{EA}+\frac{P^{2}e^{2}}{EI_{eq}}\right)dx$$

where *E* is the Young’s modulus and e is the eccentricity of the tendon. If the total strain energy is transformed to an equivalent axial strain energy, we obtained:

(6) $$\frac{1}{2}\int_{l}\left(\frac{P_{eq}{}^{2}}{EA}\right)dx=\frac{1}{2}\int_{l}\left(\frac{P^{2}}{EA}+\frac{P^{2}e^{2}}{EI_{eq}}\right)dx$$

where *P_eq_* is the equivalent axial force resultant of a simultaneous couple moment from eccentricity and axial prestressing load. Simplifying the relation above yields:

(7) $$P_{eq}=P\sqrt{1+\frac{e^{2}A}{I_{eq}}}$$

where *I_eq_* is the moment of inertia of the prestressed beam with a tendon.

From Equation (7), it is evident that the magnitude of *P_eq_* becomes larger when the eccentricity size increases and it approaches *P* while the eccentricity diminishes. Since the eccentricity induces a stiffening effect, it enhances bending stiffness and decreases geometric softening resulting from the axial load, then the effective axial load is proportional to the equivalent axial load, therefore:

(8) $$P_{eff}=\frac{P}{\sqrt{1+\frac{e^{2}A}{I_{eq}}}}$$

where *P_eff_* is the effective prestress load including the eccentricity effect, which can be replaced in Equation (3) to derive the equation of frequency:

(9) $$EI_{eq}\frac{d^{4}Y}{dx^{4}}+\frac{P}{\sqrt{1+\frac{e^{2}A}{I_{eq}}}}\frac{d^{2}Y}{dx^{2}}-\rho A\omega^{2}Y=0$$

It is the possible to write the solution of *Y*(*x*) in the form:

(10) $$Y\left(x\right)=Ae^{\lambda x}$$

where *A* is an arbitrary constant, substitution of Equation (10) into Equation (9) yields the auxiliary equation:

(11) $$\lambda^{4}+\frac{P}{EI_{eq}\sqrt{1+\frac{e^{2}A}{I_{eq}}}}\lambda^{2}-\frac{\rho A\omega^{2}}{EI_{eq}}=0$$

The roots of Equation (11) are given by:

(12) $$\lambda_{1}^{2},\lambda_{2}^{2}=-\frac{P}{2EI_{eq}\sqrt{1+\frac{e^{2}A}{I_{eq}}}}\pm \sqrt{\frac{P^{2}}{4E^{2}I_{eq}{}^{2}\left(1+\frac{e^{2}A}{I_{eq}}\right)}+\frac{\rho A\omega^{2}}{EI_{eq}}}$$

Hence, the general solution of Equation (11) can be written as:

(13) $$Y\left(x\right)=C_{1}cosh\lambda_{1}x+C_{2}sinh\lambda_{1}x+C_{3}cos\lambda_{2}x+C_{4}sin\lambda_{2}x$$

The constants *C*_1_ to *C*_4_ can be determined according to the corresponding boundary conditions of the beam. For simply supported beams, the boundary conditions are zero deflections and bending moments at both ends. This gives the natural frequency equation of a simply supported beam subjected to an eccentric axial compressive load:

(14) $$\omega_{n}={\left(\frac{\pi }{l}\right)}^{2}\sqrt{\left(\frac{EI_{eq}}{\rho A}\right)\times \left(n^{4}-n^{2}\frac{Pl^{2}}{\pi^{2}EI_{eq}\sqrt{1+\frac{e^{2}A}{I_{eq}}}}\right)}$$

where *n* is the mode number. It is clearly seen that by the increase of eccentricity, the softening influence of prestressing load declines, thereby raising the natural frequencies of prestressed beams.

## 3. Simulation of the Prestressed Beams

**Figure 2:**
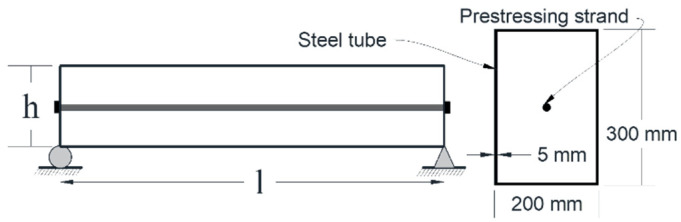
Dimensions of unbonded prestressed beam with a straight tendon.

The three-dimensional finite element method using ABAQUS software [35] has been implemented to verify the derived formula and to study the dynamic behavior of the prestressed beam. The simply supported prestressed beams with a straight strand were examined. In order to consider a high span-to-depth ratio effect, different beam lengths from 3 to 15 m were selected. The dimensions of steel tubes were 300 and 200 mm in depth and width, respectively. For the prestress tendon, a 15.7 mm in diameter wire according to the available commercial pre-stressing strand was employed, as seen in Figure 2. 

**Figure 3:**
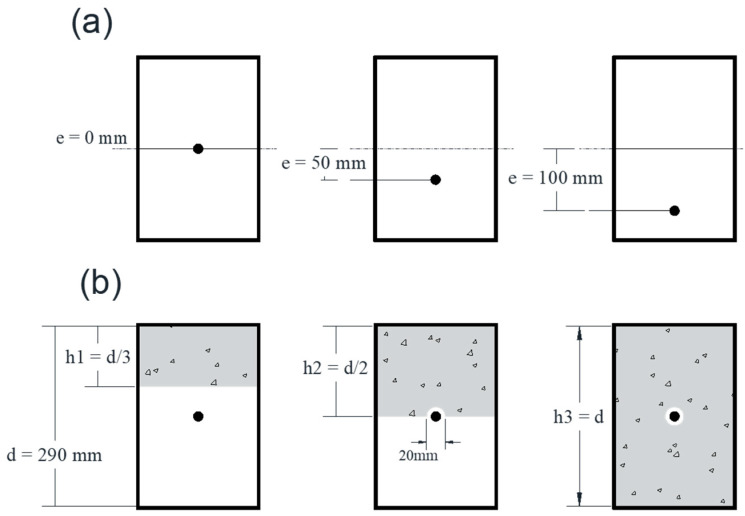
Cross-sections of simulated beams: (**a**) rectangular hollow sections (RHS) sections with different eccentricities, (**b**) concrete-filled steel tubes (CFST) cross-sections with different concrete height ratios.

A number of post-tensioned beams with a straight strand were modeled, taking the strand eccentricity and concrete height ratio into consideration. For specimens focusing on the eccentricity, RHSs with two eccentricities, one-third and two-thirds of the distance from centroid to the extreme fiber of the bottom surface, i.e., 50 and 100 mm were chosen. Regarding the concrete ratio, the concrete-filled steel tubes (CFST) were classified into three categories of h1, h2, and h3, which are one-third, half, and full proportion of inner depth (d) of RHS, respectively. A 20 mm void was considered for categories h2 and h3 to remain inserted strand free, the layout of the cross-sections is outlined in Figure 3. The mechanical properties of the steel tube, concrete, and strand were defined as linear elastic, which are shown in Table 1. The beams were pinned at both ends to simulate the simply supported boundary conditions and all rotations were kept free.

### 3.1. Prestressing Effect

**Figure 4:**
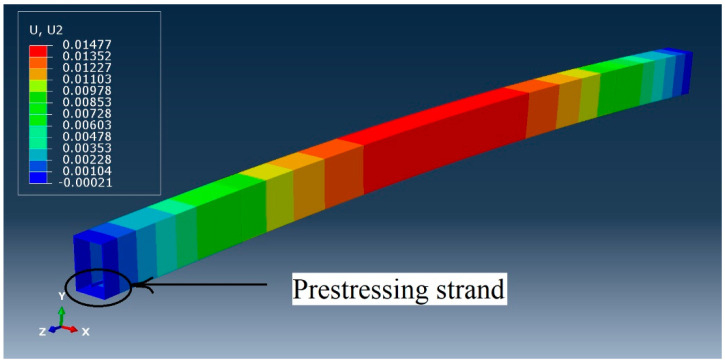
Upward deflection of the beam as a result of prestressing.

Post-tensioning load is typically simulated via either an initial strain or temperature load [36]. In this study, the latter was employed to take the prestressing effect due to initial stress. From Equation (15), the exerted temperature load can be calculated:

(15) $$T=-\frac{P}{E_{t}.A_{t}.\alpha }$$

where *T* is the temperature load used in the simulation, *P* is the prestress force, α is the thermal expansion coefficient, and *E_t_* and *A_t_* are the Young’s modulus and cross-sectional area of the strand. Four prestress load levels: 100, 200, 300, and 400 kN were calculated to evaluate the influence of load level. Figure 4 indicates upward deflection of the prestressed RHS beam with 100 mm eccentricity prior to the next step.

### 3.2. Free and Forced Vibration Analysis

**Figure 5:**
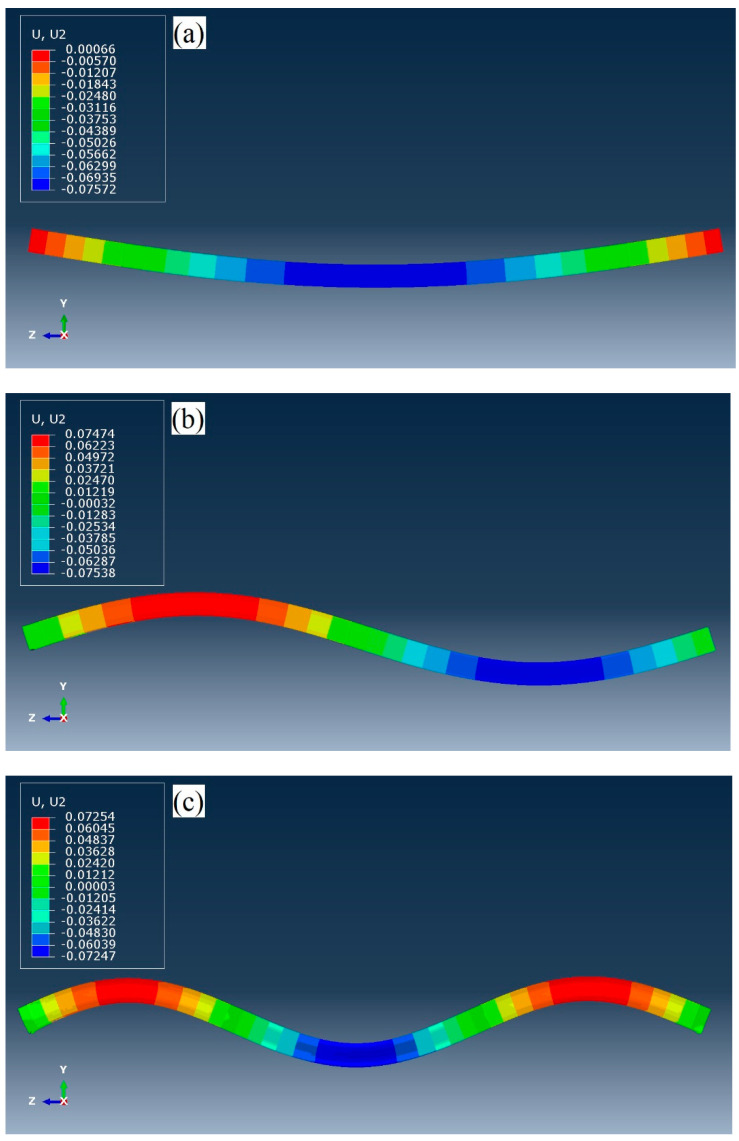
Mode shapes of unbonded prestressed beam: (**a**) 1st mode, (**b**) 2nd mode, (**c**) 3rd mode.

**Figure 6:**
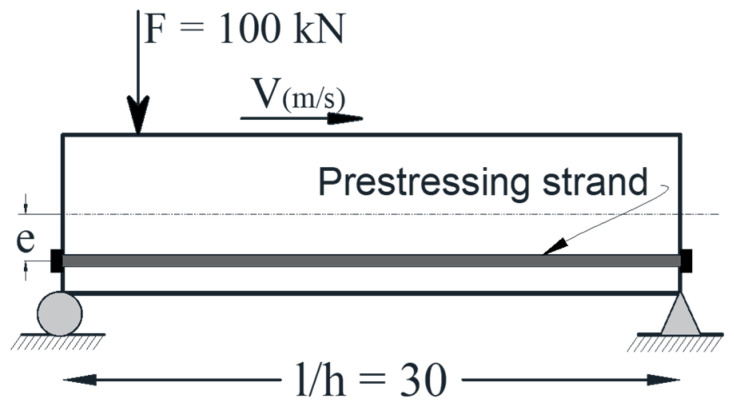
Schematic of the beam subjected to a moving load.

Concerning natural frequency and corresponding mode shape extraction, the Lanczos eigen-solver linear perturbation through which the response of analysis can only be linear was used. The material nonlinearity and applied loads are not active over a frequency analysis [35]. In the geometric nonlinearity terms, large displacement effects as a result of prestressing force were involved in the linear eigenvalue extraction. No initial imperfections were introduced to the model. The predicted first three mode shapes are shown in Figure 5. Mode shapes of the first three natural frequencies in transverse direction are similar to those of non-prestressed beams. The first mode shape comprises one half-sine wave, while mode shapes of the second and third natural frequencies include two and three half-sine waves, respectively. Regarding the forced response of post-tensioned beam subjected to a point moving load, using the implicit time integration method, different factors including velocity and excitation frequency of the load were considered. The problem was resolved by a moving load displaced with a uniform velocity along the beam length. The load excitation frequency was defined using periodic amplitude as a Fourier series. The magnitude of the load was chosen to be 100 kN. For harmonic amplitude, the cosine function along with different excitation frequencies was utilized. The time increment for the implicit scheme was selected to diverge between the minimum size of 9 × 10^−6^ and maximum size of 0.002. The nonlinear effects of large displacement were also considered. The sketch of the beam including loading and boundary conditions are presented in Figure 6.

### 3.3. Steady-State Dynamic Analysis

In a steady-state dynamic analysis, the linear response of a structure under harmonic excitation was predicted. Walsh et al. [37] conducted a damage identification method according to the alteration in the first vertical mode using frequency response functions at a given frequency range. In this study, the linear type of spacing for frequency points with the bias value of three was used to collect the response points near the frequency range. Structural damping 0.05% was selected over the whole frequency range. A concentrated nodal force was applied to the vertical displacement degree of freedom. The load was assumed to change sinusoidally with time over the range of given frequencies [35]. Although the response in this analysis was linear, nonlinear geometric effects were considered since these effects were included in the prestressing step.

### 3.4. Interactions and Element Types

**Figure 7:**
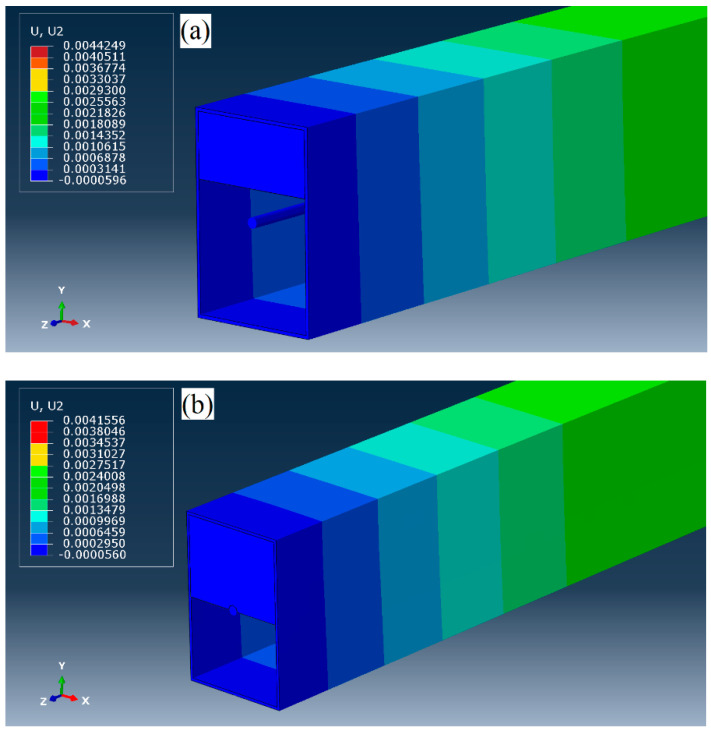
Simulated prestressed steel tube: (**a**) concrete height ratio h1, (**b**) concrete height ratio h2.

The anchorage system of the prestressing strand was modeled as the structural coupling scheme to distribute prestress load on the cross-section effectively. A surface-based contact was defined on the circumference of the tendon to allow the prestress strand slides freely and not to penetrate other surfaces. For concrete-filled specimens, a surface-based tie which is capable of quick transitions in mesh density was used to form a perfect bond behavior between the steel tube and the concrete. Figure 7 illustrates samples with concrete height ratios of h1 and h2, respectively. In relation to the point moving load, a frictionless and hard pressure–overclosure node-to-surface contact with the possibility of separation after contact was modeled between the point load and the frictionless surface of the prestressed beam. The use of a proper contact formulation between the load and the beam with large sliding is crucial [33].

**Figure 8:**
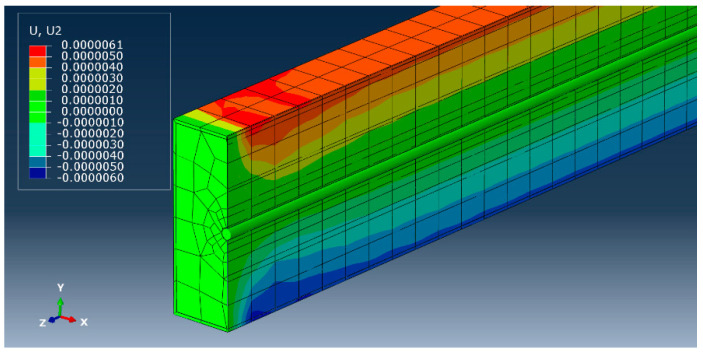
Model of meshed prestressed CFST beam.

According to the elements’ characteristics, different element types for the concrete, steel tube, and strand were used. The prestressing tendon was represented by a 2-node linear 3D truss element so that it only transmits an axial force and can bear no moment. For the concrete part, an 8-node linear brick with reduced integration and hourglass control was used. As far as the steel tube is concerned, a second-order reduced-integration brick element was employed, since this element is quite effective in bending problems and offers higher accuracy. Meanwhile, reduced integration decreases the running time and gives more precise results compared to the corresponding fully integrated elements for second-order elements [35]. The meshed cross-section view of the FEM model for the prestressed CFST beam with concrete height ratio h3 is shown in Figure 8.

### 3.5. Validation

**Figure 9:**
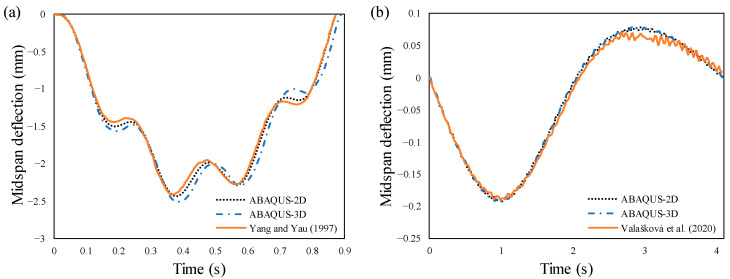
Validation of time-histories of the midspan displacement: (**a**) numerical example, (**b**) experimental test.

In order to study the convergence and estimating the FNF of the prestressed beam, three element sizes of 25, 50, and 100 mm were compared. As can be seen in Table 2, the mean values of FNFs obtained from numerical analysis are very close to those found by the mathematical equation. It is evident that CFST beams are more sensitive to the mesh size, as they have more complicated geometries. Thus, the element size 50 mm was carefully chosen for the sake of optimality and efficiency. For the moving load problem, a numerical example from Yang and Yau [25] and the test results of a moving mass on a two-span continuous beam from [38] were selected to verify the FEM simulation. The dynamic responses of the midspan deflections have been plotted in Figure 9, which are within the rational error margins with those of the reference. The small divergence is most likely resulting from the fact that our solution is a three-dimensional modeling with the possibility of separation and reconnection, whereas the reference model is a two-dimensional planner beam with continuous contact. 

## 4. Parametric Analysis

**Figure 10:**
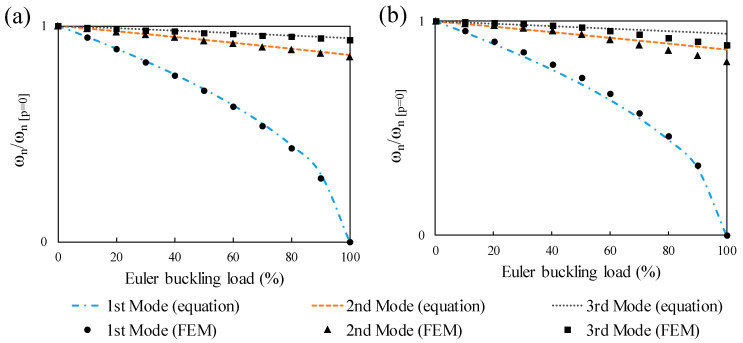
Prestress force effect on the first three natural frequencies of beams with a span-to-depth ratio of 20: (**a**) two-dimensional effects with an axial compressive load, (**b**) three-dimensional effects with a tendon.

The influence of the prestressing force, eccentricity, concrete ratio, velocity, and excitation frequency of the moving load on the dynamic behavior of the prestressed beam were investigated through a parametric analysis. Assuming the unbonded interaction between the steel strand and beam, this study neglected the influence of bonded tendon on the FNFs of the prestressed beams. Only elastic properties of the materials were considered, and based on the tubular geometry of the cross-section, the modeling was limited to the prestressed RHS with internal arrangement of the tendon. The natural frequencies were lower after post-tensioning and the reduction was mainly obvious for the first mode. The first three normalized natural frequencies of prestressed beams are given in Figure 10 in which ω_n_ represents the natural frequencies of prestressed beams with different levels of prestressing load and ω_n [p=0]_ is the natural frequencies of non-prestressed beams. The abscissa indicates the Euler buckling load proportion, which was found using:

(16) $$P_{cri}=\frac{\pi^{2}EI}{l^{2}}$$

where *P_cri_* is the lowest Euler buckling load of a simply supported beam subjected to axial load.

The results of three-dimensional modeling with prestressing strand show a minor deviation as prestressing load approaches the Euler buckling load in comparison with both two-dimensional with an axial load and conventional mechanic’s theory. This negligible difference in the three-dimensional simulation might be due to the local buckling of the RHS plates as a result of considering geometric nonlinearity while the beam is prestressed. The prestressing load has the most impact on the FNFs of the beams with different magnitudes of span-to-depth ratios and prestress forces. This effect becomes less significant for the second and third frequencies. This shows that the FNF has a higher association with prestress load. These results indicate that the first natural frequency has the most significance in practice, so only the FNF was obtained for each beam. To study the dynamic response of the prestressed beam, steel RHSs with different eccentricities and span-to-depth ratio 30 were selected.

### 4.1. Effect of Prestress Load

**Figure 11:**
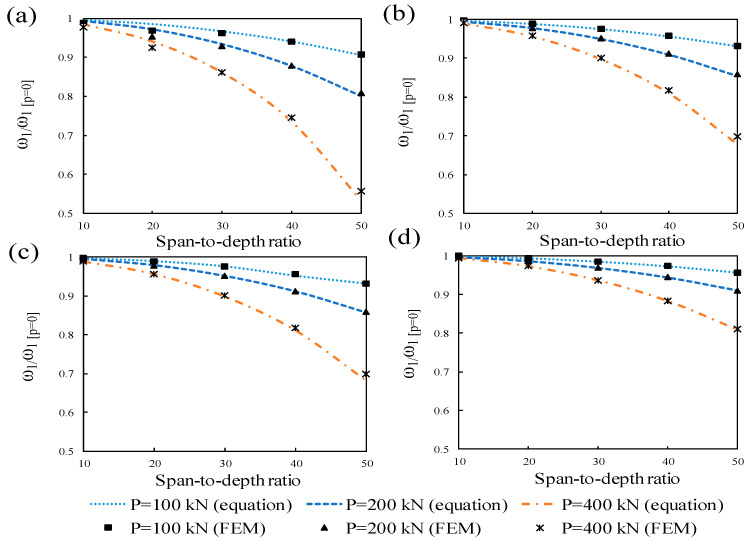
Influence of concentric prestressing force on the FNFs of beams with different span-to-depth ratios: (**a**) RHS, (**b**) specimens with concrete ratio h1, (**c**) specimens with concrete ratio h2, (**d**) specimens with concrete ratio h3.

The normalized first-order natural frequencies of the prestressed beams without eccentricity under different post-tensioning forces are given in Figure 11. In the figure, ω_1_ denotes the FNFs of prestressed beams and ω_1 [p=0]_ implies the FNFs of non-prestressed beams. The results of the frequencies of the beams obtained by the mathematical equation and simulation are in a good agreement. The FNF decreases gradually with the increase of the prestress force and this reduction became more apparent through a rising span-to-depth ratio. It was evident that following an increase of the span-to-depth ratio, the slenderness ratio grew, and consequently resulted in a slenderer beam. In such a case, the impact of prestressing load on the beam escalates and it lessens the bending stiffness of the beam. As a result, the FNFs of the beams are reduced.

**Figure 12:**
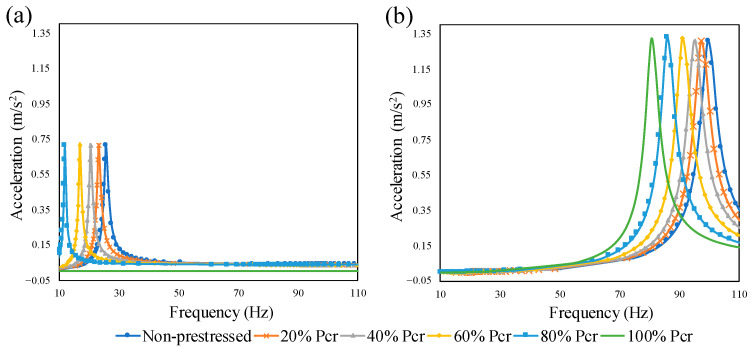
Influence of the concentric prestressing force on the peak frequency of the beam: (**a**) first mode, (**b**) second mode.

**Figure 13:**
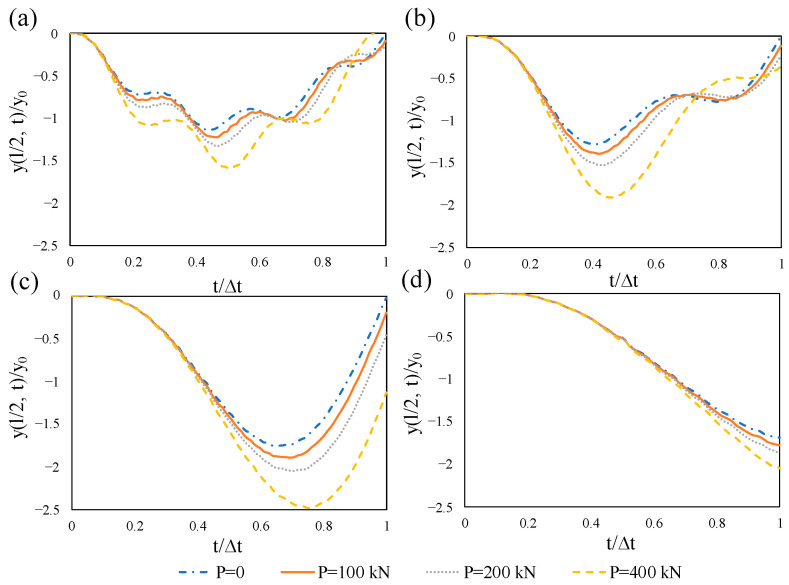
Influence of concentric prestressing force on the normalized midspan deflection of the beam subjected to a moving load with different velocities: (**a**) *v* = 25 m/s, (**b**) *v* = 50 m/s, (**c**) *v* = 100 m/s, (**d**) *v* = 200 m/s.

The frequency response functions for the first two modes were plotted in terms of acceleration and frequency in Figure 12. Six different levels of prestress load from zero to the full proportion of the Euler buckling load were selected for the beam. The prestressing effect was characterized through the alteration in frequency peak. As is evident, the peak frequency shifts to the left as prestress force approaches the buckling load. Figure 12a also illustrates that this effect is more significant for the first mode so that no peak occurs for the beam when the prestress load is equal to the buckling load (horizontal green line). The time-histories for the normalized midspan displacement of the beam were computed and the results are illustrated in Figure 13. In the figure, y0 is the static midspan displacement resulting from the point load at the midspan, y(l/2, t) is the midspan displacement at the time t, and ∆T is the time required for the moving point load to pass through the beam. It can be clearly seen in Figure 13 that in the presence of the prestress force, for higher values of the axial force, the maximum response of the beam increases. This happens because of the “compression softening” effect in which the prestress load softens the beam. It should be noted that at a given speed of the moving load, by increasing the level of the axial load, the maximum response occurs slightly later. For the prestress load 400 kN and velocity 200 m/s, which is unexpected and similar to an impact, the maximum response occurs after the load leaves the beam.

**Figure 14:**
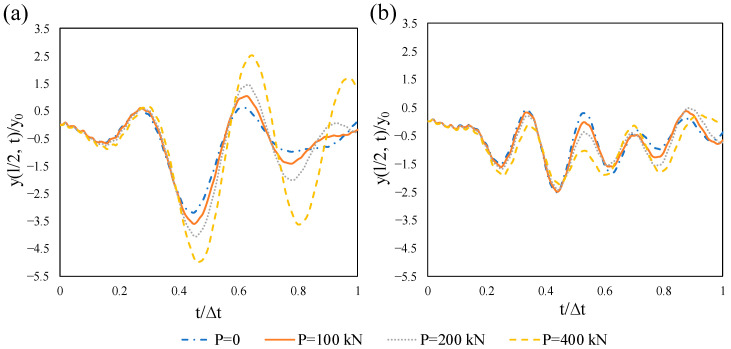
Influence of concentric prestressing force on the normalized midspan deflection of the beam subjected to a harmonic moving load with a constant velocity of 25 m/s: (**a**) Ω = 40 rad/s, (**b**) Ω = 100 rad/s.

Dynamic response of the simply supported prestressed beam under a moving cosine harmonic load with a constant velocity was studied. The effect of the prestressing load for two excitation frequencies Ω = 40 and Ω = 100 rad/s is given in Figure 14. The first natural frequencies of the beam with prestressing loads of 0, 200, and 400 kN are 70.2, 65.5, and 60.5 rad/s, respectively. Therefore, two excitation frequencies were chosen with about one half and three halves of the fundamental frequency of the non-prestressed beam so that they are considerably lower and higher than that. Once the excitation frequency was 40 rad/s, the compressive axial force noticeably augmented the dynamic midspan deflection. The higher the prestress force is, the larger the midspan deflects (Figure 14a). This is comparable to the case without an excitation frequency in which the compressive force decreases the flexural stiffness. As mentioned earlier, the midspan displacement surges incrementally with the increase of axial force. However, when the load frequency is higher than the fundamental frequency, the sensitivity of the beam’s dynamic deflection to the prestress force is insignificant (Figure 14b).

### 4.2. Effect of Eccentricity

**Figure 15:**
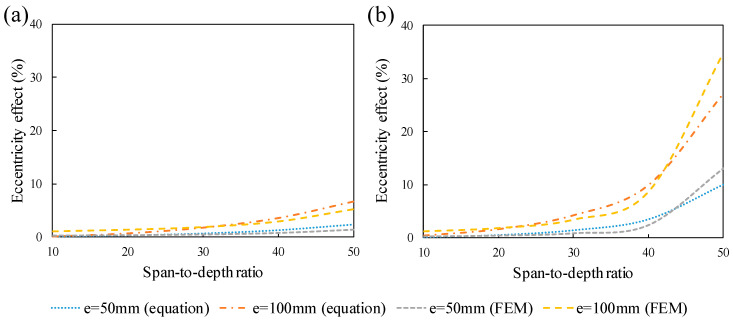
Increase of the FNFs of prestressed beams due to eccentricity: (**a**) P = 200 kN, (**b**) P = 400 kN.

In order to study the effect of the eccentricity on the fundamental frequency of the eccentrically prestressed beam, a formula was derived from the equation of motion and effective load obtained from strain energy. The precision of the natural frequency formulation was verified by comparison with the FEM results. It is worth noting that the accuracy of the simulation was previously verified compared to the conventional mechanical solution. The fundamental frequency variations for different ratios of length to depth are revealed in Figure 15. To calculate the eccentricity effect, relative differences were computed as:

(17) $$\mathrm{Eccentricity}\;\mathrm{effect}\;\left(\%\right)=\frac{\omega_{1\;\left[e=i\right]}-\omega_{1\;\left[e=0\right]}}{\omega_{1\;\left[e=0\right]}}\times 100$$

where *i* is the eccentricity values, which are 50 and 100 mm here. As shown in Figure 15, for specimens with a 50 mm eccentric tendon, the first natural frequency increases slightly and this effect is more obvious for beams with larger spans. Clearly, the increase of eccentricity enlarges the increase of the fundamental frequency so that it plays a more stiffening role. It can be realized that eccentricity improves the bending stiffness and deteriorates the softening effect, which is a result of an axial load; however, this effect is very slight for small eccentricities. It can be observed in Figure 15 that the FNF tends to increase as eccentricity increases and the higher amount of prestressing force intensifies this phenomenon. Comparison between the values of the eccentricity effect from FEM and the derived formula clarifies that the formula works well and it can accurately predict the effect of eccentricity with an acceptable error margin.

**Figure 16:**
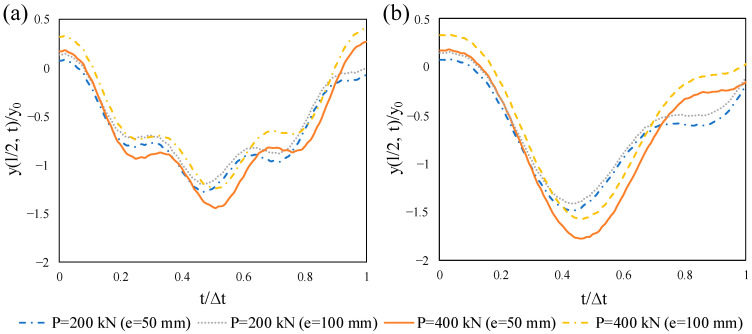
Influence of eccentricity on the normalized midspan deflection of the prestressed beam subjected to a moving load: (**a**) *v* = 25 m/s, (**b**) *v* = 50 m/s.

The influence of the eccentricity of the prestressing tendon on the dynamic midspan deflection of the beam under a moving load was examined. In the presence of eccentricity, prestressing procedure results in upward deflection, which is clearly perceived in Figure 16. It is noticeable that after the moving load arrival, this deflection that is above the zero line moves to the negative zone. Regardless of the moving speed, the maximum response gradually decreases by raising the eccentricity and this is due to the fact that the eccentricity reduces axial force effect. It was noted that the internal stresses caused by the moving load can be reduced by the application of the eccentricity. The eccentricity of the prestress strand does not change the dynamic response of the beam subjected to harmonic moving load meaningfully, so the related figures are not given herein. This was also concluded by Şimşek and Kocatürk [32].

### 4.3. Effect of Concrete Ratio

The CFST beam is widely used for its excellent structural performance, though the concrete cracks in tension zone are its weaknesses. The prestressing technique is a promising method in order to deal with this flaw [39]. However, in this study, microcracks were not considered and all concrete parts were perfect and uncracked. Different ratios of concrete height were nominated to investigate the concrete effect on the fundamental frequency of beams with various length to depth ratios. Dealing with a CFST beam, its effective flexural stiffness can be calculated as the sum of flexural stiffness of the steel tube and the concrete:

(18) $${\left(EI\right)}_{eff}=E_{s}I_{s}+E_{c}I_{c}$$

where (*EI*)*_eff_* is the effective bending stiffness of the CFST beam, *E_s_*, *E_c_*, *I_s_*, and *I_c_* are the Young’s modules and the moment of inertia of steel and concrete, respectively. The mentioned method is useful for the filled tube with concrete ratio h3, whereas for the other two categories h1 and h2, this method is not useful since these sections are not symmetric. Therefore, the method of transformed section treating the steel elastic modulus as reference material behavior was employed. The effective properties of composite sections are tabulated in Table 3. The natural frequencies can be determined by using the effective EI and mass per length.

**Figure 17:**
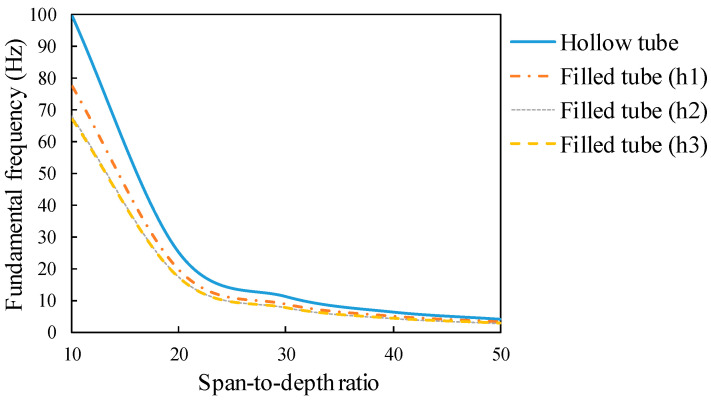
Influence of concrete height ratio on the FNF of the non-prestressed beam.

It can obviously be seen in Figure 17 that the hollow tube has the largest fundamental frequency among all sections. By comparing the moment of inertia from Table 3, this can mainly be seen because the RHS section is lighter in weight than filled tubes. It was also found out that half and full concrete-filled tubes have almost similar FNFs. The section with concrete ratio h1 has nearly the same bending stiffness as the section with concrete ratio h2, nonetheless, it presents a higher FNF. This means that this section is lighter and more sustainable, which can be taken into account for designing the beam profile.

### 4.4. Effect of Velocity

**Figure 18:**
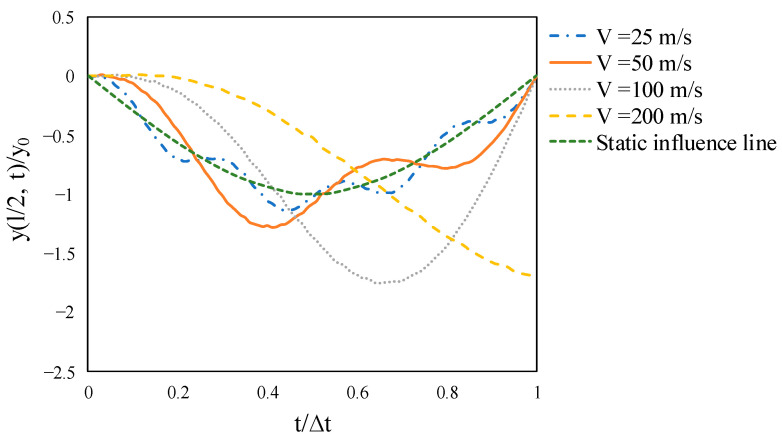
Time-histories for normalized midspan displacement of the non-prestressed beam subjected to a moving load with various velocities.

This section studies the dynamic response of the simply supported prestressed beam subjected to a moving point load with various velocities and excitation frequencies. The effect of velocity on the normalized midspan deflection of a beam having the critical velocity of 200 m/s under a moving point load is depicted in Figure 18. The critical velocity can be calculated by v_cr_ = Lω1/π in which ω1 is the first natural frequency of the beam. The variation of velocity has a substantial effect on the dynamic midspan displacement. The moving load with velocities 25, 50, 100, and 200 m/s results in the maximum midspan vertical displacement 0.1348, 0.1520, 0.2090, and 0.2016 at load positions 0.43, 0.41, 0.65, and 1, respectively. It is clear that as the velocity grows, the maximum deflection tends to diverge more from the static influence line and shift to the right. Except for the critical velocity at which its dynamic response is at maximum, the midspan deflections of all other velocities are zero when the load exits the beam.

The maximum response ratios, henceforth called the dynamic magnification factor, is defined as the maximum ratio of the dynamic and static midspan displacements. The dynamic magnification factor relies on the velocity parameter, which is a function of load speed and the FNF of the beam. Table 4 lists the dynamic magnification factors for the prestressed beam with different post-tensioning forces and velocities. It is obvious that the axial force causes an increase in the dynamic magnification factor and it is also sensitive to the velocity of the moving load. The implication may result from the decrease in the flexural stiffness of the beam as a result of the softening effect. It is noteworthy to mention that beyond the speed of 125 m/s, the dynamic magnification factors start to decrease, which are in agreement with the results obtained by Olsson [24].

**Figure 19:**
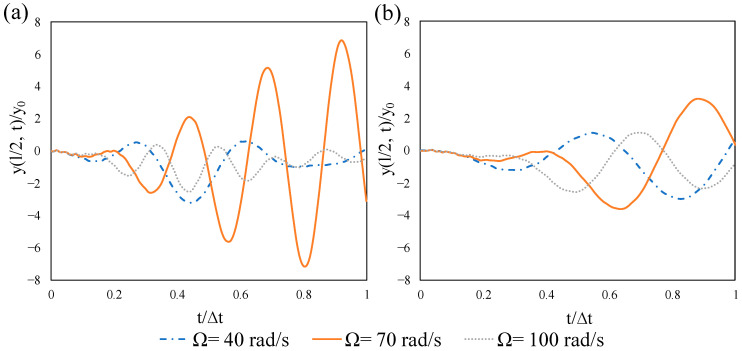
Time-histories for normalized midspan displacement of the non-prestressed beam subjected to different values of frequencies: (**a**) *v* = 25 m/s, (**b**) *v* = 50 m/s.

The effect of the velocity and excitation frequency of the moving point load is discussed here by taking two velocities with frequencies 40, 70, and 100 rad/s, as shown in Figure 19. As mentioned earlier, the FNF of the non-prestressed beam is 70.2 rad/s. Figure 19a illustrates that the maximum dynamic response of the beam is remarkably higher for the frequency of 70 rad/s, which clarifies the resonance effect. However, this effect is reduced by the increase of the velocity, as can be seen in Figure 19b. The dynamic magnification factors for Ω = 70 rad/s and velocities 25 and 50 m/s are 7.11 and 3.576, respectively. This shows the contribution of the velocity to the resonance, which is more important at a slower pace.

## 5. Conclusions

In order to examine the relationship between the prestress force with the fundamental natural frequency and dynamic characteristics of the large-span beam, two approaches based on a conventional mechanics theory and numerical simulation were employed. Derived from the strain energy theory and equation of motion, a formula was proposed for calculation of the natural frequencies comprising the eccentricity effect. Theoretical and numerical cases were then presented which discuss the difference between the first vertical natural frequencies of the beams influenced by the eccentric prestress force. The parametric studies were performed taking into consideration the effect of the axial compressive load, eccentricity, concrete ratio, and velocity and excitation of the moving load on the dynamic response of the beam.

The main conclusions are drawn as follows:

1. The fundamental frequency of the large-span beam is affected in the presence of a prestressing load. However, it depends on the size of the load and slenderness ratio of the beam. On the condition that either the prestress load or span-to-depth ratio is large enough, the first natural frequency declines due to the “compression softening” effect.
2. Relating to the CFST beams, the effective mass and moment of inertia play an important role in the weight and stiffness of the structure. The cross-sections filled by one-third and half of the inner depth represent nearly identical bending stiffness levels while the beam with concrete ratio h1 is lighter. Thus, this fact may be considered for a sustainable design process.
3. The pre-tensioning force results in a greater dynamic response of the large-span beam under a moving point load; however, this reduction tends to disappear due to an increasing load excitation frequency. Correspondingly, the dynamic magnification factor rises by an increment in the prestress load, which clarifies a direct connection.
4. The results indicated that the eccentricity causes more flexural stiffness and reduces the softening effect resulting from the prestress force. Accordingly, in comparison with the beams subjected to concentric prestressing, the fundamental frequency grows.
5. Due to the fact that eccentricity induces initial upward deflection, the dynamic vertical displacement decreases; however, this effect for beams under harmonic load is meaningless. It should be noted that the dynamic midspan displacement increases as the velocity of the moving point load rises to about 62% of the critical velocity of the beam, then it begins to decrease.

It is strongly recommended that for beams with large span-to-depth ratios, the application of eccentricity is advantageous so as to reduce the softening effect caused by the prestressing system.

It is noted that a prestressed steel RHS beam is not similar to a prestressed concrete beam where the strands are bonded to the surrounding concrete. Further research is needed to comprehend exactly how the bonded tendons and prestressing level alter the FNFs of slender and stocky CFST beams since based on the above-mentioned results, the FNFs of prestressed beams with unbonded tendons are reliant on the size of the load and slenderness ratio of the beam to behave consistently with the “compression-softening” theory.

## Figures and Tables

**Table 1:** The mechanical properties of the material.

Material	Modulus of Elasticity (GPa)	Poisson Ratio	Density (kg/m^3)^	Thermal Expansion Coefficient
Steel tube	200	0.3	7850	-
Strand	200	0.3	7850	1.0 × 10^−5^/°C
Concrete	35	0.2	2420	-

**Table 2:** Comparison among the fundamental natural frequencies (FNFs)of prestressed beams.

Span to Depth Ratio	Concrete Height Ratio	Prestress Load (kN)	Equation (Hz)	Element Size					
				100 mm		50 mm		25 mm	
				ABAQUS (Hz)	ABAQUS/Equation	ABAQUS (Hz)	ABAQUS/Equation	ABAQUS (Hz)	ABAQUS/Equation
20	h3	0	17.066	16.416	0.962	16.802	0.985	16.876	0.989
20	h3	400	16.586	15.940	0.961	16.336	0.985	16.411	0.989
20	h2	0	17.552	16.807	0.958	17.156	0.977	17.203	0.980
20	h2	400	16.782	16.063	0.957	16.427	0.979	16.478	0.982
20	h1	0	19.697	18.979	0.964	19.195	0.975	19.237	0.977
20	h1	400	18.827	18.153	0.964	18.378	0.976	18.424	0.979
20	N/A	0	25.139	24.675	0.982	24.664	0.981	24.651	0.981
20	N/A	400	23.659	23.283	0.984	23.272	0.984	23.260	0.983
Mean			-	-	0.967	-	0.980	-	0.983
S.D.			-	-	0.011	-	0.004	-	0.004

**Table 3:** Effective properties of all cross-sections.

Cross-Section	Concrete Height (mm)	* Moment of Inertia (cm^4^)	Mass per Length (kg/m)
Hollow tube	N/A	6384	38.465
Filled tube (h1)	96.67	8448	82.913
Filled tube (h2)	145	8510	105.136
Filled tube (h3)	290	13,100	171.8

* Moment of inertia about major axis corresponding to the steel elastic modulus.

**Table 4:** The dynamic magnification factor of unbonded prestressed beam for various velocities and prestress loads.

Velocity (m/s)	Prestress Load (kN)			
	0	100	200	400
25	1.1333	1.2236	1.3286	1.5973
50	1.2783	1.3959	1.5286	1.9098
75	1.6144	1.7525	1.9133	2.3450
100	1.7571	1.8885	2.0479	2.4835
125	1.7853	1.9165	2.0633	2.4366
150	1.7590	1.8735	2.0125	2.3790
200	1.6947	1.7823	1.8735	2.0511

## Data Availability

The FE data used to support the findings of this study are available upon request.
